# ScopeNovo: a *de novo* peptide sequencing method using spectrum–spectrum similarity to refine the prediction accuracy

**DOI:** 10.1093/bioinformatics/btag755

**Published:** 2026-10-15

**Authors:** Zhi Song, Zejian Ding, Lusheng Wang

**Affiliations:** Department of Computer Science, City University of Hong Kong, Hong Kong, China; Department of Computer Science, City University of Hong Kong, Hong Kong, China; Department of Computer Science, City University of Hong Kong, Hong Kong, China

## Abstract

**Motivation:**

*De novo* peptide sequencing from tandem mass spectra is essential for characterizing unknown proteins, identifying novel post-translational modifications, and analysing samples without reference databases. While recent deep learning models have improved the predicted peptide accuracy, they typically select the predicted peptide sequence based solely on sequence-level decoding scores. Very often, high scores are assigned to peptides without the direct support from the observed mass spectrum.

**Results:**

We present “ScopeNovo”, a *de novo* peptide sequencing method that improves predicted peptide accuracy by using spectrum-spectrum similarity between the input spectrum and the “representative” spectrum corresponding to the predicted peptide sequence. ScopeNovo generates multiple candidate peptides using an existing peptide *de novo* sequencing learning model and reranks candidates based on spectrum–spectrum similarity. To test our method, we use π-PrimeNovo as the base learning model. Experimental results on the standard nine-species benchmark datasets $V_{1}$ and $V_{2}$ show that ScopeNovo can substantially improve the prediction accuracy. We designed two settings, where Setting 1 is for well-annotated species, and Setting 2 is for species that are not well annotated. For Setting 1, ScopeNovo improves the average peptide accuracies of π-PrimeNovo over the nine species by 6.1% and 4.0% on $V_{1}$ and $V_{2}$, respectively. For Setting 2, ScopeNovo can still improve the peptide accuracy of π-PrimeNovo over the nine species by 3.4% and 1.9% on the $V_{1}$ and $V_{2}$ benchmark datasets, respectively. Compared to the best-known method, RankNovo, the peptide accuracies over the nine species on $V_{1}$ and $V_{2}$ of our method for Setting 1 are 0.711 and 0.737, where the peptide accuracies for RankNovo are 0.687 and 0.682. Even for Setting 2, the peptide accuracies of our method are comparable to those of RankNovo.

**Availability and implementation:**

ScopeNovo is implemented in Python under an MIT license. The source code is freely available at https://github.com/SoSongzhi/ScopeNovo.git. The test data are available at https://doi.org/10.5281/zenodo.23181841.

## 1 Introduction

*De novo* peptide sequencing based on tandem mass spectrometry (MS/MS) is a core technique in proteomics for characterizing proteins. It is the first and key step for sequencing unknown proteins such as monoclonal antibodies. Given an MS/MS spectrum *x*, machine learning models predict a protein peptide sequence *y* by learning a distribution $p_{\theta }(y|x)$ to overcome the limitations of classical database search (Perkins *et al.* 1999), propose novel sequences, and handle post-translational modifications (PTMs). Lots of deep learning methods have been proposed in the past few years and the whole area has been reshaped. The pioneer work started with DeepNovo (Tran *et al.* 2017), which coupled convolutional and recurrent neural networks with local dynamic programming to predict peptide sequences directly from tandem mass spectra. Casanovo (Yilmaz *et al.* 2024) subsequently recast this task as sequence-to-sequence translation, using an autoregressive transformer to improve prediction accuracy. Within this framework, Eloff et al. *(*2025) developed InstaNovo to further advance autoregressive sequencing performance, whereas InstaNovo + introduced diffusion-based refinement to iteratively correct preliminary predictions and reduce error propagation. More recently, Zhang et al. (2025) proposed π-PrimeNovo, which replaced autoregressive decoding with a non-autoregressive transformer to predict all residues in parallel, thereby limiting sequential error accumulation and achieving superior amino acid-level accuracy.

In parallel with these advances in sequence modeling, recent studies have also sought to improve how tandem mass spectra are represented and learned. Specifically, Qiao et al. (2021) developed PointNovo, which represents spectra as irregular point clouds, preserving high-resolution mass measurements while avoiding information loss caused by m/z discretization. GraphNovo adopts a graph-based formulation, using graph neural networks to mitigate missing fragmentation and guide peptide-sequence prediction through spectral-peak graph modeling (Mao *et al.* 2023). Beyond spectral representation, Xia et al. (2024) introduced AdaNovo, which leverages a conditional-mutual-information-based adaptive training strategy to improve robustness against biased and noisy spectra. ContraNovo leverages contrastive learning to better align mass-spectral and peptide-sequence embedding spaces (Jin *et al.* 2024). Sanders *et al.* (2025) further extended this line of development through Cascadia, a transformer-based model for *de novo* peptide sequencing from DIA mass spectrometry data, improving generalization across instruments and experimental protocols.

Despite learning model architectural progress, candidate peptide sequence selection remains overwhelmingly sequence-centric. By maximizing sequence likelihood, models often assign high probabilities to sequence hallucinations whose implied spectra poorly match the observed MS/MS signal. This symptom is particularly prevalent for noisy spectra, long peptides, and rare PTMs. Furthermore, independent transformer decoding ignores the natural redundancy of LC–MS/MS experiments, where near-duplicate spectra of the same peptide are generated during elution (Frank *et al.* 2008, Griss *et al.* 2016, The and Käll 2016). To make use of the near-duplicate spectra of the same peptide, lots of post-processing tools like Percolator (Käll *et al.* 2007) or rerankers such as pNovo3 (Yang *et al.* 2019), NovoRank (Seo *et al.* 2025), and RankNovo (Qiu *et al.* 2025) have been developed.

Percolator introduced semi-supervised discriminative rescoring for peptide–spectrum matches, showing that calibrated post-search scoring can substantially improve confidence estimation in database-based peptide identification (Käll *et al.* 2007). pNovo3 used pDeep-predicted spectra and a learning-to-rank framework to rerank candidate peptides, demonstrating that agreement between predicted and observed fragment intensities can help distinguish similar sequence candidates (Yang *et al.* 2019). NovoRank explicitly combines spectral clustering with deep learning, using both cluster-derived proteomic features and individual-spectrum features to rerank candidates generated by existing *de novo* tools (Seo *et al.* 2025). The state-of-the-art method RankNovo reranks candidates from multiple base models by stacking them as a multiple-sequence alignment and applying axial attention, scoring each candidate via predicted peptide- and residue-level mass-deviation metrics (Qiu *et al.* 2025).

In this article, we introduce ScopeNovo, a model-agnostic reranking method that reports the most likely peptide sequence based on a list of candidate peptide sequences for an input mass spectrum generated from a learning model. The key of our reranking method is to build a peptide–spectrum library based on a set of annotated spectra, where each peptide sequence may correspond to multiple mass spectra. The latent vector of a spectrum can be obtained as the output vector of the encoder in the well-trained learning model. We then use the *latent spectrum–spectrum similarity*, which is the cosine similarity of the two latent vectors of the corresponding spectra, to rerank the candidate peptide sequence. Since each peptide sequence may correspond to multiple spectra in the library, we will calculate the average latent spectrum–spectrum similarity of the top three spectra of each peptide sequence and use this score to rerank.

Experimental results on the standard nine-species benchmark (Tran *et al.* 2017) datasets $V_{1}$ and $V_{2}$ show that ScopeNovo can substantially improve the prediction accuracy. *Setting 1* is designed to handle the spectra for well-annotated species. In this idealized scenario, ScopeNovo improves the average peptide accuracy of π-PrimeNovo over the nine species by 6.1% and 4.0% on $V_{1}$ and $V_{2}$, respectively. It also achieves notable gains on challenging species, e.g. 7.8% and 9.2% on $V_{1}$ and $V_{2}$ for *C. bact*, *Setting 2* is designed to test the case, where species are not well annotated. This is more important for new species, where little is known. Under this scenario, ScopeNovo can still improve the peptide accuracy of π-PrimeNovo over the nine species by 3.4% and 1.9% on the $V_{1}$ and $V_{2}$ benchmark datasets, respectively. Compared to the best known *de novo* sequencing model, RankNovo, the peptide accuracies over the nine species on $V_{1}$ and $V_{2}$ of our method for Setting 1 are 0.711 and 0.737, where the peptide accuracies for RankNovo are 0.687 and 0.682. Even for Setting 2, the peptide accuracies of our method are comparable to those of RankNovo.

The experiment on Setting 1 shows that annotated spectral library can really help to improve the predicted peptide accuracy. This work finds a new application for generating simulated spectra with input peptide sequence and new simulation tools for generating annotated spectral library are required to help peptide de novo sequencing.

## 2 Materials and methods

### 2.1 Overview

We introduce ScopeNovo, a model-agnostic reranking method that reports the most likely peptide sequence based on a list of candidate peptide sequences for an input mass spectrum generated from a learning model. The key of our reranking method is to build a peptide–spectrum library based on a set of annotated spectra, e.g. the training dataset of a learning model, where each peptide sequence may correspond to multiple mass spectra. The *latent vector* of a spectrum can be obtained as the output vector of the encoder in the well-trained learning model. We then use the *latent spectrum–spectrum similarity*, which is the cosine similarity of the two latent vectors of the corresponding spectra, to rerank the candidate peptide sequence. Since each peptide sequence may correspond to multiple spectra in the library, we will calculate the average latent spectrum–spectrum similarity of the top three spectra of each peptide sequence and use this score to rerank.

In particular, ScopeNovo contains three steps.

#### 2.1.1 Step 1: generating candidate peptide sequences

Both autoregressive and non-autoregressive learning methods take a mass spectrum as input, and give a peptide sequence as the predicted sequence with the highest probability within their search space. Autoregressive models typically do a heuristic beam search to report the peptide. Non-autoregressive model such as π-PrimeNovo tries to find a path in the output probability matrix as the predicted peptide. We can see that very often the ground truth peptide sequence is among the top-*k* candidates with highest probability, but its probability is not the highest.

ScopeNovo is model-agnostic. It can use any learning model to generate a list of top-*k* candidate peptide sequences. To illustrate its advantages, we use π-PrimeNovo to generate k=100 candidate peptides. Concretely, we run beam search directly on the log-probability matrix $P\in R^{L\times V}$ emitted by its non-autoregressive decoder, keeping 100 paths and expanding each with its top-*M* tokens (M=10) per position, while pruning any branch that exceeds the observed precursor mass during decoding. We then filter the resulting 100 candidates to those matching the precursor mass within a 0.1 Da tolerance, and additionally include the single highest-scoring mass-feasible path obtained by π-PrimeNovo’s precise mass control (PMC) dynamic-programming unit.

#### 2.1.2 Step 2: constructing peptide–spectrum library

We construct a peptide–spectrum library based on a set of annotated spectra, e.g. the training dataset of a learning model, where each peptide sequence may correspond to multiple mass spectra.

#### 2.1.3 Step 3: reranking candidate peptide using latent spectrum–spectrum similarity

Given an input spectrum $s={\left\{(m_{i},I_{i})\right\}}_{i=1}^{N}$ containing *N* peaks, where $m_{i}$ and $I_{i}$ denote the mass/charge value and intensity of the *i*th peak, respectively, we obtain its spectrum-level *latent vector* from the spectrum encoder of the well-trained learning model, e.g. π-PrimeNovo (see Fig. 1a). Specifically, each mass/charge value is first encoded by a sinusoidal encoding function:


$$E_{\text{mass}}(m_{i},j)=\{\begin{array}{cc} \;\text{sin}\;\left(2\pi \frac{m_{i}}{\rho_{\text{min}}{\left(\frac{\rho_{\text{max}}}{\rho_{\text{min}}}\right)}^{\frac{2j}{d}}}\right), & j\leq \frac{d}{2},\\ \;\text{cos}\;\left(2\pi \frac{m_{i}}{\rho_{\text{min}}{\left(\frac{\rho_{\text{max}}}{\rho_{\text{min}}}\right)}^{\frac{2j}{d}-1}}\right), & j>\frac{d}{2}, \end{array}$$

for j=1,…,d, where $\rho_{\text{min}}=0.001$ and *ρ*_max_ = 10 000, which define the wavelength range. The corresponding intensity value is projected into the same *d*-dimensional space as


$$E_{\text{intensity}}(I_{i})=W_{I}I_{i},$$

where $W_{I}\in R^{d\times 1}$ is a learnable projection matrix. The embedding of the *i*th peak is then obtained by element-wise summation:


$$v_{i}=E_{\text{mass}}(m_{i})+E_{\text{intensity}}(I_{i}).$$

**Figure 1:**
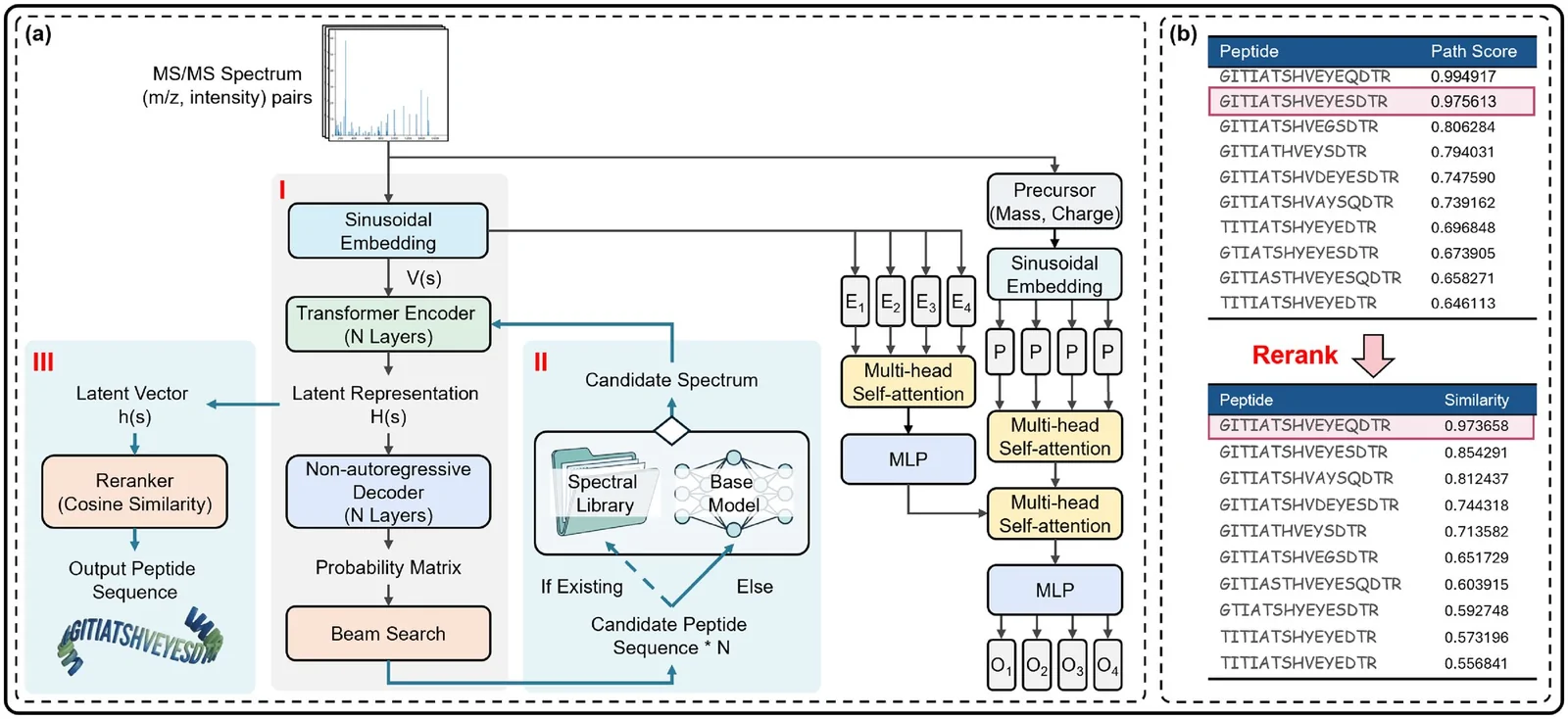
ScopeNovo overview: spectrum-consistent *de novo* decoding via latent-space reranking. (a) Given an observed MS/MS spectrum *x* (peaks and precursor metadata), we use π-PrimeNovo spectrum encoder to produce a latent representation and a non-autoregressive decoder generates a high-accuracy top-*k* peptide candidate set via wide-beam decoding. For each candidate peptide *y*, ScopeNovo associates an auxiliary spectrum $\tilde{x}$ by retrieving a reference spectrum from a spectral library. Both *x* and $\tilde{x}$ are embedded by the same spectrum encoder, and the cosine similarity of their embeddings defines a spectrum-consistency score used to rerank candidates at inference time. (b) Illustration of the reranking effect on a real case: candidates with high sequence scores but weak spectral support are demoted, while spectrally consistent candidates are promoted to top-1.

The peak representation matrix $V(s)=[v_{1},\ldots ,v_{N}]$ of the *N* peaks is passed through the transformer-based spectrum encoder, yielding a contextualized latent representation $H(s)=[h_{1},\ldots ,h_{N}]$. Note that the number *N* of peaks for different mass spectra may differ. Though padding is used to handle the number of peaks, directly using the cosine similarity of padded H(s) will lead to lower similarity for spectra corresponding to the same peptide sequence. Thus, the *latent vector* of the spectrum is defined as the mean of the *N* vectors $h_{i}$,


(1) $$h(s)=\frac{1}{N}\sum_{i=1}^{N}h_{i},$$

which is used to represent the input mass spectrum.

Let *s* and $\tilde{s}$ be two spectra. The *latent spectrum–spectrum similarity* is defined as the cosine similarity of the two latent vectors of the two corresponding spectra,


(2) $$\mathrm{sim}(s,\tilde{s})=\frac{h{\left(s\right)}^{⊤}h(\tilde{s})}{\|h(s){\|}_{2}\|h(\tilde{s}){\|}_{2}},$$

where $h{\left(s\right)}^{⊤}$ is the transpose of the vector, $\|h(s){\|}_{2}$ is the length of the vector. For each candidate peptide sequence *p* in the list of candidate peptides generated by a learning model, e.g. π-PrimeNovo, if *p* is in the peptide–spectrum library, we will compute the latent spectrum–spectrum similarity between the input mass spectrum and the spectrum corresponding to the candidate peptide. When multiple mass spectra exist for a candidate peptide, we use the average latent spectrum–spectrum similarity of the top-3 spectra. If the candidate peptide is not in the peptide–spectrum library, the latent spectrum–spectrum similarity is set to be 0. We then report the peptide with the highest average latent spectrum–spectrum similarity as the predicted peptide. When no candidate peptide in the list is found in the peptide–spectrum library, we use π-PrimeNovo’s original prediction.

### 2.2 Candidate peptide generation for autoregressive and non-autoregressive models

For an autoregressive model, the prediction of the *i*-token is based on the input mass spectrum as well as the first i−1 predicted tokens. It uses beam search to report the candidate peptide with the highest probability within the search space. Here, we extend the beam search algorithm to report at most K=100 candidate peptides with the highest probabilities such that the total mass of the peptide is equal to the precursor mass of the input mass spectrum.

For a non-autoregressive model such as π-PrimeNovo, it outputs an L×V probability matrix, where L=40 is the maximum length of the predicted peptide sequence, and *V* is the number of amino acids (including modifications). At each position we preselect the top-*M* tokens (with M=10) and perform beam expansion via tensorized score composition, with only one top-*M* operation per step. To enforce physical validity, we apply a precursor-mass constraint during decoding: we track the accumulated mass of each partial peptide hypothesis, and prune all branch expansions that cannot match the observed precursor mass within a 0.1 Da tolerance. We finally report at most 100 candidates using the same 0.1 Da precursor-mass tolerance.

### 2.3 Peptide–spectrum library construction

A key observation for the training datasets is that a peptide often corresponds to multiple mass spectra. Moreover, the mass spectra corresponding to the same peptide may differ a lot even if they are generated from the same instrument and have the same precursor charge state (see Fig. 2, available as supplementary data at *Bioinformatics* online). A classic method to measure the similarity of two mass spectra is to use the *modified cosine* similarity (Watrous *et al.* 2012). For peptide NQTAEKEEFEHQQK in the Mouse split of the nine-species-V1 dataset, there are 12 corresponding mass spectra with the same precursor charge state 2+. The average pairwise modified cosine similarity among the spectra is only 0.466, while the average pairwise latent cosine similarity is 0.875 (see Figs 1 and 2, available as supplementary data at *Bioinformatics* online). Thus, we believe that mass spectra corresponding to the same peptide have something in common and the output latent representation h(s) of the encoder provides a more accurate and reliable depiction of the common properties. Therefore, the latent spectrum–spectrum similarity of the two latent vectors is used to select the final candidate peptides.

The peptide–spectrum library is obtained from the training dataset. Each entry in the library is a pair (pep,list), where *pep* is the peptide sequence and *list* contains a list of mass spectra corresponding to the same peptide. The number of mass spectra in the list is set to be at most 10. When more than 10 mass spectra exist, we use the farthest first strategy to select 10 spectra that are far away from each other.

Given a candidate peptide *p* of an input spectrum *s*, we search the library to find the spectra list of *p*, without restricting the reference spectra to the same precursor charge state as the input spectrum. For each spectrum $s_{i}$ in the list, we compute the spectrum–spectrum similarity $s(v(s),v(s_{i}))$ between the two latent vectors v(s) and $v(s_{i})$ and use the average of the top-3 $s(v(s),v(s_{i}))$’s as the score to select the predicted peptide.

## 3 Results

### 3.1 Datasets

We evaluate ScopeNovo on the standard nine-species high-resolution benchmark (Tran *et al.* 2017), using the publicly reprocessed release (MSV000090982). MS/MS spectra are searched against the corresponding reference proteome with a target–decoy strategy, and high-confidence peptide–spectrum matches are retained at 1% PSM-level FDR.

### 3.2 Main results

We evaluate ScopeNovo on the standard nine-species high-resolution benchmark in two different settings. *Setting 1 (External Library)* performs spectrum-consistency reranking against a high-quality external library aggregated from the nine-species training datasets and MassIVE-KB (∼2–3 M curated spectra; see Section 3.1) it represents the upper-bound performance attainable when abundant external reference spectra are available. *Setting 2 (Self-Constructed Library)* addresses the realistic case where such external resources are limited: it constructs the reference database entirely from in-batch high-confidence predictions of the base *de novo* model on the test cohort itself, with no external annotation, and reranks every test spectrum against this self-constructed database; a strict cross-species filter is applied during retrieval to prevent data leakage, together with a high-confidence threshold t=0.95 tuned on a held-out validation split. Table 1 reports amino-acid precision and peptide accuracy for all nine species on both nine-species-V1 and nine-species-V2 datasets.

**Table 1:** Comparison of *de novo* peptide sequencing methods on nine-species-V1 and nine-species-V2 benchmarks.^a^

	Nine-species-V1										Nine-species-V2									
Methods	Bac.	C.b.	Hon.	Hum.	M.m.	Mou.	R.b.	Tom.	Yea.	Avg.	Bac.	C.b.	Hon.	Hum.	M.m.	Mou.	R.b.	Tom.	Yea.	Avg.
Amino acid precision																				
Casanovo	0.788	0.676	0.708	0.676	0.754	0.765	0.761	0.792	0.756	0.743	**0.923**	0.787	0.776	0.856	0.828	0.806	0.854	0.874	0.865	**0.881**
Casanovo V2	0.801	0.687	0.721	0.687	0.767	0.774	0.768	0.802	0.768	0.754	0.843	0.799	0.788	0.865	0.836	0.813	0.867	0.882	0.875	0.865
InstaNovo	0.802	0.656	0.718	0.691	0.756	0.742	0.794	0.774	0.767	0.745	0.852	0.751	0.799	0.847	0.848	0.634	0.894	0.864	0.877	0.850
ContraNovo	0.828	0.700	0.764	0.778	0.798	0.803	0.804	0.812	0.787	0.785	0.863	0.802	0.817	**0.909**	0.864	0.830	0.903	0.881	0.883	0.865
AdaNovo	**0.858**	**0.798**	0.767	0.690	0.804	0.778	**0.878**	0.818	0.791	0.798	0.792	0.816	0.756	0.800	0.804	0.757	0.759	0.831	0.801	0.794
RankNovo	0.807	0.771	**0.880**	**0.784**	**0.838**	**0.823**	0.876	**0.846**	**0.828**	**0.827**	0.836	**0.836**	**0.891**	0.836	**0.879**	**0.920**	0.896	**0.893**	0.879	0.880
π -PrimeNovo	0.840	0.715	0.767	0.725	0.799	0.797	0.823	0.822	0.795	0.789	0.874	0.806	0.824	0.870	0.869	0.808	0.921	0.885	0.891	0.866
**ScopeNovo (Setting 1)**^b^	0.849	0.715	0.778	0.729	0.811	0.809	0.816	0.825	0.796	0.797	0.885	0.822	0.839	0.884	0.882	0.841	0.931	0.897	0.900	0.885
**ScopeNovo (Setting 2)**	0.844	0.722	0.774	0.732	0.806	0.809	0.829	0.828	0.798	0.795	0.877	0.814	0.829	0.879	0.874	0.821	**0.926**	0.889	**0.892**	0.868
Peptide accuracy																				
Casanovo	0.621	0.437	0.494	0.446	0.568	0.485	0.593	0.622	0.601	0.551	0.687	0.522	0.579	0.662	0.656	0.522	0.645	0.730	0.738	0.682
Casanovo V2	0.630	0.445	0.504	0.455	0.579	0.493	0.600	0.629	0.607	0.559	0.693	0.532	0.587	0.670	0.661	0.531	0.654	0.737	0.743	0.683
InstaNovo	0.652	0.459	0.535	0.515	0.595	0.525	0.635	0.635	0.618	0.582	0.704	0.512	0.625	0.670	0.666	0.504	0.679	0.721	**0.758**	0.698
ContraNovo	0.688	0.486	0.580	**0.625**	0.633	0.574	0.678	0.674	0.673	0.628	0.709	0.533	0.615	**0.759**	0.698	0.567	0.722	0.729	0.725	0.701
AdaNovo	0.753	**0.638**	0.620	0.494	0.671	0.546	**0.797**	0.684	0.660	0.660	0.636	**0.624**	0.597	0.590	0.641	0.472	0.538	0.693	0.653	0.635
RankNovo	**0.765**	0.592	0.642	0.621	0.688	0.595	0.765	0.720	**0.726**	**0.687**	0.713	0.603	0.617	0.717	0.712	0.559	0.744	0.724	0.680	0.682
π -PrimeNovo	0.725	0.532	0.602	0.575	0.654	0.572	0.701	0.701	0.703	0.650	0.712	0.572	0.612	0.710	0.714	0.548	0.766	0.719	0.684	0.697
**ScopeNovo (Setting 1)**^b^	0.789	0.610	0.665	0.628	0.731	0.660	0.755	0.752	0.744	0.711	0.748	0.664	0.669	0.762	0.772	0.664	0.816	0.771	0.702	0.737
**ScopeNovo (Setting 2)**	0.749	0.572	**0.643**	0.624	**0.690**	**0.637**	0.733	**0.732**	0.721	0.684	**0.728**	0.619	**0.641**	0.750	**0.743**	**0.611**	**0.788**	**0.745**	0.691	**0.716**

a For ScopeNovo we report two operating regimes: Setting 1 (external library, spectrum-consistency reranking against a curated external spectral library), which characterizes the upper-bound performance attainable when high-quality external spectral resources are available; and Setting 2 (self-constructed library), in which the reference library is bootstrapped entirely from high-confidence in-batch *de novo* predictions on the test data itself, placing ScopeNovo under the same resource assumptions as the baselines and yielding a direct comparison. In each column, the best value among the baselines and ScopeNovo (Setting 2) is in bold; ScopeNovo (Setting 1) is reported separately for reference and excluded from the bold comparison.

b Setting 1 evaluates ScopeNovo with spectrum-consistency reranking against an external library on both V1 and V2; Setting 2 evaluates ScopeNovo with spectrum-consistency reranking against a self-constructed reference database built from in-batch high-confidence predictions, also on both V1 and V2. Bac. = *Bacillus*, C.b. = *C. bact*, Hon. = *Honeybee*, Hum. = *Human*, M.m. = *Methanosarcina mazei*, Mou. = *Mouse*, R.b. = *Rice Bean*, Tom. = *Tomato*, Yea. = *Yeast*.

#### 3.2.1 Setting 1: well-annotated species

To handle the spectra for well-annotated species, we construct a peptide–spectrum library in Setting 1, which is curated from two complementary sources: (i) the nine-species training dataset and (ii) MassIVE-KB (Wang *et al.* 2018).

We evaluate ScopeNovo against seven representative baselines spanning autoregressive decoders (Casanovo, Casanovo V2, InstaNovo, ContraNovo, AdaNovo), reranking-based methods (RankNovo), and the non-autoregressive base π-PrimeNovo, on both the nine-species-V1 and nine-species-V2 benchmarks under two metrics: peptide accuracy and amino-acid precision. Across all nine species and the average column, ScopeNovo (Setting 1) attains 0.711 average peptide accuracy on V1 and 0.737 on V2, exceeding the best non-ScopeNovo baseline by +2.4 pp on V1 (over RankNovo, 0.687) and +4.0 pp on V2 (over π-PrimeNovo, 0.697). Compared with the base π-PrimeNovo decoder for Setting 1, our method yields +6.1% peptide accuracy improvement on V1 (0.650→0.711) and +4.0% improvement on V2 (0.697→0.737) on average peptide accuracy, with consistent gains on all nine species on each benchmark. The details are given in Table 1.

Note that the library used in Setting 1 contains the curated testing species datasets (the nine species); of course, predicted mass spectra are not included. When no candidate peptide in the candidate list is found in the peptide–spectrum library, we directly use π-PrimeNovo’s original prediction. This setting is useful when we need to predict peptides for mass spectra from well-annotated species. The next setting is designed to handle spectra from species that are not well annotated.

#### 3.2.2 Setting 2: species that are not well-annotated

In order to test the prediction accuracy for species that are not well annotated, we use Setting 2, where the testing datasets (the nine species) are removed from the initial peptide–spectrum library. That is, the initial peptide–spectrum library only contains MassIVE-KB (Wang *et al.* 2018).

Let *T* denote the set of mass spectra from such a target species, for which we aim to predict peptide sequences. To make use of the abundant in-batch redundancy in *T*, we first run the π-PrimeNovo decoder over *T* to obtain an initial peptide prediction for every spectrum. The sets of predicted peptide sequences whose π-PrimeNovo confidence probability is ≥0.95 (with peptide accuracies of 97.90% and 96.28% on nine-species-V1 and nine-species-V2) are added into the initial peptide–spectrum library to form the *second-phase library*, against which ScopeNovo performs spectrum-consistency reranking.

Despite operating without any in-domain reference spectra, ScopeNovo in Setting 2 still yields consistent gains over the base decoder: +3.4 pp average peptide accuracy on V1 (0.650→0.684) and +1.9 pp on V2 (0.697→0.716), and attains the best peptide accuracy on four of nine species on V1 and six of nine species on V2—most notably on *Honeybee*, *Methanosarcina mazei*, *Mouse*, and *Tomato*. Compared with the strongest reranker RankNovo, Setting 2 is within 0.3 pp on V1 (0.684 versus 0.687, where reranking has largely saturated), and exceeds RankNovo by +3.4 pp on V2 (0.716 versus 0.682) on all nine species. The details are given in Table 1. Most importantly, when compared with Setting 1 (which has access to all nine species’ curated reference spectra), Setting 2 closes roughly half of the gap to Setting 1’s average peptide accuracy on both V1 (0.684 versus 0.711) and V2 (0.716 versus 0.737), while requiring no in-domain peptide–spectrum dataset at all. This demonstrates that the spectrum–spectrum similarity refinement method is still effective and broadly applicable to the realistic case of poorly annotated species.

#### 3.2.3 Peptide accuracy–coverage analysis based on model confidence

Figure 3, available as supplementary data at *Bioinformatics* online, presents peptide accuracy–coverage curves for π-PrimeNovo, RankNovo, ScopeNovo (Setting 1 and Setting 2). All predictions are ranked in descending order according to π-PrimeNovo model confidence, and coverage is progressively increased by including predictions from highest to lowest confidence. At each coverage level, peptide accuracy is computed on the retained subset. Under this protocol, this curve reflects whether reranking can correct predictions on spectra with progressively lower π-PrimeNovo confidence.

As shown in Fig. 3a, available as supplementary data at *Bioinformatics* online, on the nine-species-V1 benchmark, ScopeNovo (Setting 1) consistently attains the highest accuracy–coverage curves across nearly all species, while ScopeNovo (Setting 2) also remains above the base π-PrimeNovo model for most of the coverage range, with particularly clear gains on *C. bact*, *Honeybee*, *M. mazei*, *Mouse*, and *Tomato*. Figure 3b, available as supplementary data at *Bioinformatics* online, shows a similar trend on nine-species-V2: ScopeNovo (Setting 1) again yields the strongest overall curves, and ScopeNovo (Setting 2) continues to outperform π-PrimeNovo on most species, including *C. bact*, *Honeybee*, *Human*, *M. mazei*, *Mouse*, and *Tomato*. Importantly, these gains persist as coverage approaches 1.0, where spectra with lower π-PrimeNovo confidence are gradually incorporated and the curves converge to the full-dataset accuracies reported in Table 1. Together, these results show that spectrum-consistency reranking can correct a substantial fraction of errors made by the base model, including predictions originating from spectra assigned relatively low confidence by π-PrimeNovo.

#### 3.2.4 Inference efficiency

The total inference time on the nine-species-V1 (314 571 spectra) using a single NVIDIA V100S-32GB GPU is reported in Table 2. In general, our method is faster than Casanovo and ContraNovo, while it is slower than π-PrimeNovo. Taking the total time across all species for example, ScopeNovo (Setting 2) finishes in 12.19 h, compared with 3.83 h for the non-autoregressive base π-PrimeNovo, and 33.26 h and 99.11 h for the autoregressive baselines Casanovo and ContraNovo. ScopeNovo is thus 2.7×/8.1× faster than the two autoregressive baselines while achieving substantially higher accuracy.

**Table 2:** End-to-end inference time for different methods on nine-species-V1 dataset, with ScopeNovo evaluated under Setting 2.^a^

Species	Casa. (h)	ContraN. (h)	π **-Prime. (h)**	Scope. (h)
Bac.	6.30	18.55	0.73	2.12
C.b.	3.83	10.47	0.42	1.21
Hon.	6.60	18.99	0.83	2.32
Hum.	3.04	9.15	0.34	1.25
M. m.	3.16	8.32	0.39	1.28
Mou.	0.69	1.74	0.08	0.64
R.b.	0.74	3.82	0.09	0.34
Tom.	6.05	19.26	0.65	2.15
Yea.	2.85	8.81	0.30	0.88
Total	33.26	99.11	3.83	12.19

a Bac. = *Bacillus*, C.b. = *C. bact*, Hon. = *Honeybee*, Hum. = *Human*, M.m. = *Methanosarcina mazei*, Mou. = *Mouse*, R.b. = *Rice bean*, Tom. = *Tomato*, Yea. = *Yeast*. Casa. = Casanovo, π-Prime. = π-PrimeNovo, Contra. = ContraNovo, Scope. = ScopeNovo.

#### 3.2.5 Understanding the impact of peaks

Theoretical peaks for a peptide sequence are the mathematically predicted mass-to-charge (*m*/*z*) values of ion fragments that should appear if a given peptide sequence is broken apart in a mass spectrometer. A peak in mass spectrum corresponds to a theoretical peak of known peptide sequence if the mass difference of the two peaks is within a range [−δ,δ], where δ is the peak error tolerance and δ=0.1 Da. Note that, mass spectra have lots of noise peaks as well as peaks corresponding to theoretical peaks of the peptide sequence. To understand how these two kinds of peaks affect the *de novo* sequencing process, we conduct an ablation study on the Mouse split of the nine-species-V1 benchmark by removing some percentages of non-theoretical peaks and theoretical peaks, respectively, and observe the corresponding peptide accuracies. Figure 5a, available as supplementary data at *Bioinformatics* online, shows the results for removing non-theoretical peaks. The blue and red bars represent the performances of π-PrimeNovo and ScopeNovo, respectively. The green bars represent the performance of a modified ScopeNovo version, where instead of using latent spectrum–spectrum similarity, we use the modified cosine similarity (Watrous *et al.* 2012). We can see that when non-theoretical peaks are removed from 10% to 50%, the performances of the three methods slightly decrease. This shows that the non-theoretical peaks have a minor impact on the performance. Figure 5b, available as supplementary data at *Bioinformatics* online, shows the results for removing theoretical peaks. We can see that when 10% of theoretical peaks are removed, the performances of all three methods decrease slightly. When 20% of theoretical peaks have been removed, the performances of the three methods drop dramatically.

### 3.3 Library dependence analysis

A key concern is how much of ScopeNovo’s performance gain depends on the availability of empirical spectra in the peptide–spectrum library. We analyse this dependence on the Mouse test dataset. We randomly remove some items in the peptide–spectrum library to observe the cases, when the library contains 20%, 50%, 80%, and 100% of the items. Table 3 shows that the peptide accuracy degrades gracefully and the improvement of ScopeNovo with respect to π-PrimeNovo (with peptide accuracy 0.572) is still 0.8% with only 20% coverage, and the gap to full-coverage performance narrows as coverage increases. This suggests that peptide–spectrum libraries do help, and moderate coverage (e.g. 50%) already yields substantial gains of 3.8%.

**Table 3:** Peptide accuracy with varying library coverage on the Mouse test set.^a^

Method	Library coverage			
	20%	50%	80%	100%
ScopeNovo	0.580	0.610	0.640	0.660
Improvement	+0.8%	+3.8%	+6.8%	+8.8%

a Note that π-PrimeNovo has peptide accuracy 0.572.

### 3.4 Combined with other transformer models

A key advantage of ScopeNovo is its model-agnostic design: it can be applied to both autoregressive and non-autoregressive transformer-based models that provide a spectrum encoder. Other than π-PrimeNovo, we also combine ScopeNovo with the other two autoregressive transformer models, Casanovo V2 and InstaNovo. For each model, we use its pretrained spectrum encoder to generate the latent embedding vectors for both observed and reference spectra. The candidate generation step uses the original model’s native decoding strategy (beam search for Casanovo V2, the diffusion-based decoder for InstaNovo) with some minor changes. All other hyperparameters (K=100, L=10, k=3) remain the same. Table 4 shows that ScopeNovo can be combined with both non-autoregressive and autoregressive transformer models and the combined versions consistently improve the peptide accuracy across all three base models. Using the Mouse test set, we can see that the improvements in peptide accuracy for Casanovo V2, InstaNovo, and π-PrimeNovo are 4.9%, 8.9%, and 8.8%, respectively.

**Table 4:** ScopeNovo can also improve the peptide accuracy for other transformer models.^a^

Base model	Acc. of mod.	Acc. of combined
Casanovo V2	0.493	0.542 (+4.9%)
InstaNovo	0.525	0.614 (+8.9%)
π -PrimeNovo	0.572	0.660 (+8.8%)

a The predicted peptide accuracies on the Mouse test set for different base models, Casanovo V2, InstaNovo, and π-PrimeNovo, are given in “acc. of mod.” column. The accuracies for different models combined with ScopeNovo are given in the “acc. of combined” column.

The consistent improvement across different models demonstrates that ScopeNovo is not tied to a specific base model’s inductive biases. The method requires only that the base model provides a spectrum encoder whose latent space captures peptide-relevant spectral features. This makes ScopeNovo a versatile post-processing tool for the de novo sequencing community.

### 3.5 Evaluation on non-tryptic digestion data

To further evaluate the generalizability of ScopeNovo beyond tryptic data, we evaluate ScopeNovo and other baseline models on PXD009737-GluC, a non-tryptic *Human* dataset generated by Sun *et al.* (2018) and reanalysed by π-MSNet (Dai *et al.* 2026). PXD009737-GluC is generated using GluC digestion and contains longer peptide sequences. It therefore differs substantially from the training data of π-PrimeNovo and the nine-species benchmarks. Using the peptide–spectrum library built from MassIVE-KB and nine-species-V1 (not including the non-tryptic Human dataset), ScopeNovo achieves the highest peptide accuracy 44.70% among all evaluated methods, outperforming RankNovo (third best) and InstaNovo (second best) by 3.03% and 1.97%, respectively. ScopeNovo also improves the peptide accuracy of the base π-PrimeNovo model from 40.77% to 44.70% and the amino-acid precision from 57.93% to 58.66%. See Table S2, available as supplementary data at *Bioinformatics* online, for details.

## 4 Discussion

We present ScopeNovo, a *de novo* peptide sequencing method that improves predicted peptide accuracy by using spectrum–spectrum similarity between the input spectrum and the “representative” spectrum corresponding to the predicted peptide sequence. The experiment on Setting 1 shows that annotated spectral library can really help to improve the predicted peptide accuracy. This work shows the possibility that new simulation tools for generating annotated spectral library based on peptide sequences may help its dual task of predicting the peptide sequence based on an input spectrum. We used Prosit (Gessulat *et al.* 2019) to generate simulated mass spectra on the nine-species-V1 benchmark dataset. Mouse is the only species for which incorporating Prosit-generated spectra improves the prediction accuracy. The main reason is that the simulated spectra generated by Prosit contain only a limited number of peaks corresponding to theoretical b-ion and y-ion peaks of the peptide sequence, while “hidden” useful peaks (used by the learning model) are not included. Prosit is originally designed to improve the search methods, where most algorithms focus on matching of theoretical peaks. See “Source Attribution of Successful Corrections” in Supplementary Material, available as supplementary data at *Bioinformatics* online.

## Supplementary Material

btag755_Supplementary_Data
