# Supplementary material for: ScopeNovo: a de novo peptide sequencing method using spectrum–spectrum similarity to refine the prediction accuracy
Source: Bioinformatics. 2026 Oct 15;42(10):btag755. doi: 10.1093/bioinformatics/btag755 (PMC13682837; doi:10.1093/bioinformatics/btag755)
Supplement: Supplementary file 1 [file btag755_supplementary_data.pdf]

Supplementary Information

ScopeNovo: a De Novo Peptide Sequencing Method Using Spectrum-Spectrum Similarity to Refine the Prediction Accuracy

Zhi Song[1,†], Zejian Ding[1,†], Lusheng Wang[1,2,*]

[1]*Department of Computer Science, City University of Hong Kong, Hong Kong, China*

[2]*City University of Hong Kong Shenzhen Research Institute, Guangdong, China*

[†]These authors contributed equally to this work.

E-mail: cswangl@cityu.edu.hk

1 Spectra corresponding to the same peptide can exhibit weak peak-wise similarity

As discussed in the Peptide-Spectrum Library Construction Section, the peptide `NQTAEKEEFEHQQK` from the Mouse split of the 9-species-V1 benchmark corresponds to 12 spectra with the precursor charge state 2+. To illustrate the weak peak-wise similarity among the spectra, we show a representative pair of experimental MS/MS spectra. Figure S1 shows spectra F4:4114 and F4:4121, whose modified cosine similarity is only 0.0309, whereas their latent cosine similarity is 0.967. Despite corresponding to the same peptide, the two spectra differ substantially in their peak patterns.

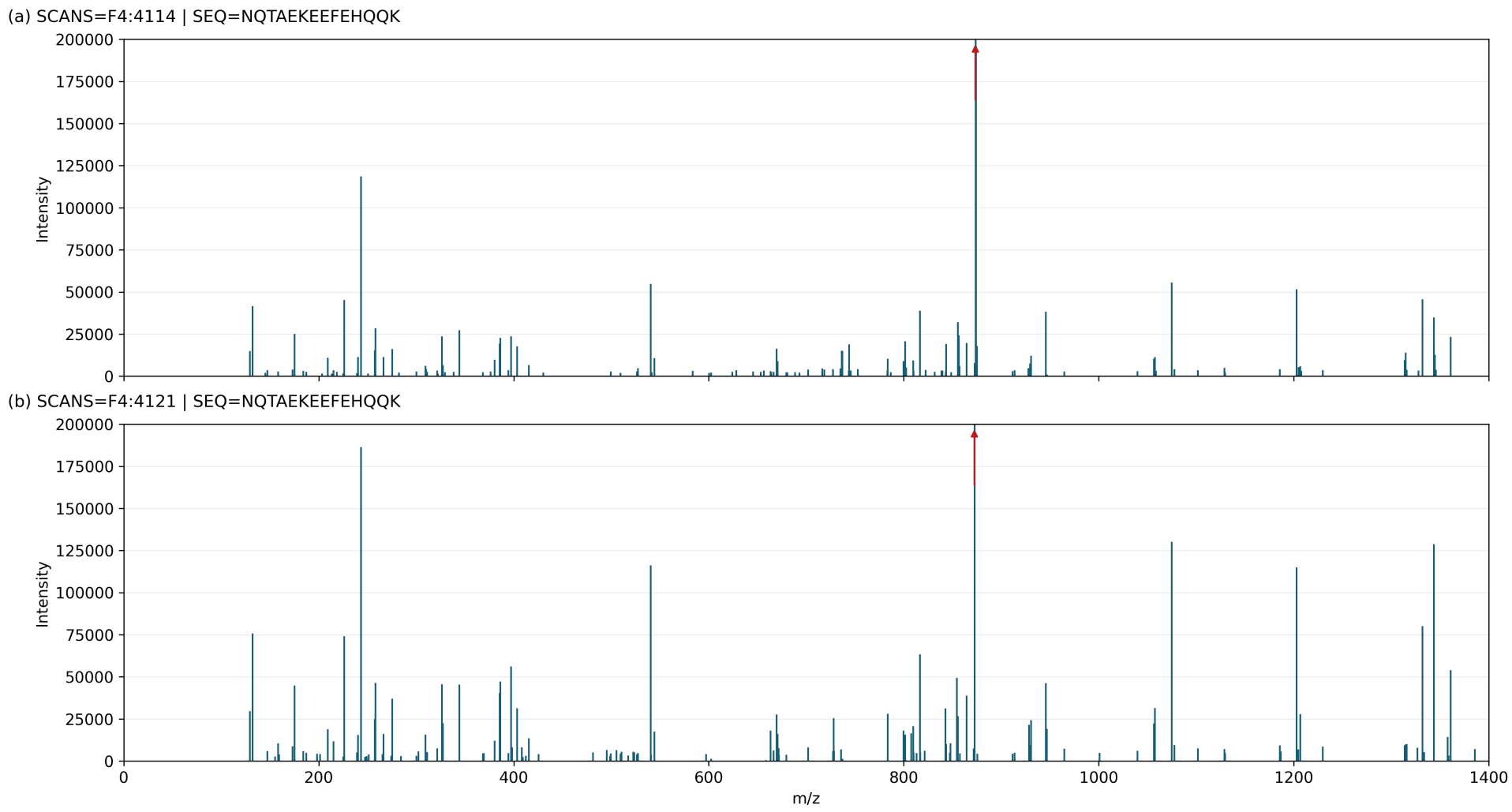

Figure S1: Two MS/MS spectra corresponding to the peptide `NQTAEKEEFEHQQK` in the Mouse split of the 9-species-V1 benchmark: (a) spectrum F4:4114 and (b) spectrum F4:4121. Although both spectra correspond to the same peptide, the spectra show very weak peak-wise agreement, with a modified cosine similarity of 0.0309. Red arrows indicate peaks whose intensities exceed the plotted y-axis limits (200,000) and are therefore truncated.

To determine whether this observation extends beyond the representative pair, we further compare the similarity distributions of all 12 spectra corresponding to `NQTAEKEEFEHQQK`. All pairwise comparisons among these spectra are used to form the same-peptide group, resulting in 66 spectrum pairs. For comparison, we randomly select 12 spectra with precursor charge state 2+ from different peptides, yielding 144 different-peptide spectrum pairs when paired with the 12 target spectra. For each pair, we compute both modified cosine similarity and latent cosine similarity. As shown in Fig. S2, the same-peptide pairs have a median modified cosine similarity of 0.242, compared with 0.020 for the different-peptide pairs. In the latent space, the corresponding median similarities are 0.942 and −0.121, respectively. The modified cosine similarities of the same-peptide pairs also show a broader distribution, whereas the latent similarities are more concentrated. These results indicate that the latent vector captures shared properties among spectra corresponding to the same peptide more consistently than modified cosine similarity, supporting its use for candidate reranking in ScopeNovo.

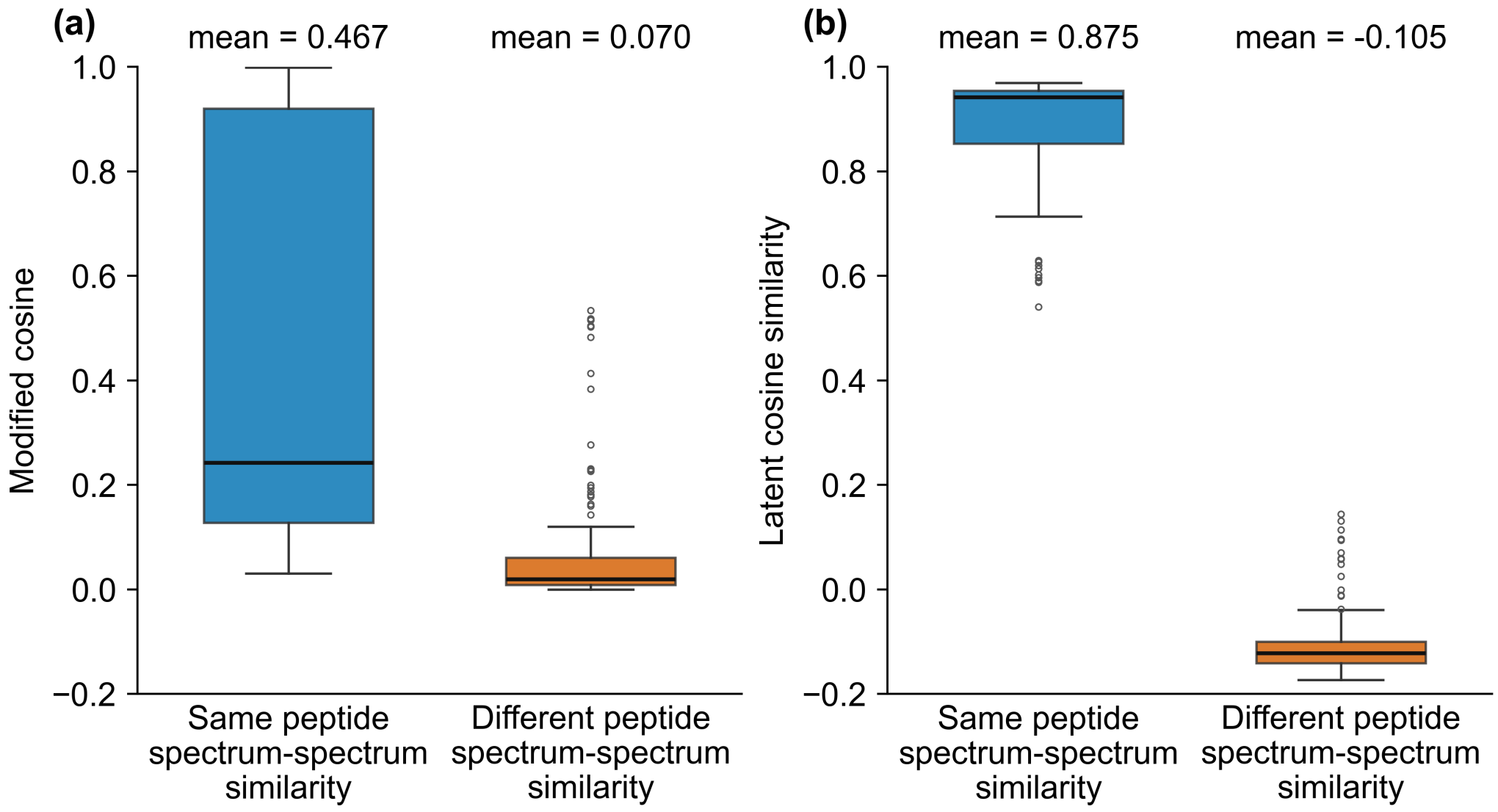

Figure S2: Distribution of spectrum–spectrum similarities for spectra corresponding to the same and different peptide sequences. (a) Modified cosine similarity, with median values of 0.242 and 0.020 for same-peptide and different-peptide spectrum pairs, respectively. (b) Latent cosine similarity, with median values of 0.942 and −0.121 for same-peptide and different-peptide spectrum pairs, respectively.

2 Annotation quality of the self-constructed library in Setting 2

Because Setting 2 constructs its reference library entirely from π-PrimeNovo's own high-confidence ($\geq$ 0.95) predictions on the test cohort, we validate the annotation quality of this self-constructed library by measuring the ground-truth accuracy of the incorporated high-confidence predictions. For every test spectrum whose π-PrimeNovo confidence is at least 0.95, we compare the predicted peptide with the ground-truth peptide. Table S1 shows that the high-confidence predictions used to build the library are correct for 97.90% of the spectra on 9-species-V1 and 96.28% on 9-species-V2, confirming that the self-constructed library provides largely reliable spectrum.

Table S1: Peptide accuracy of the high-confidence predictions used to construct the self-constructed peptide–spectrum library in Setting 2.

9-species-V1		9-species-V2	
Species	Peptide accuracy (%)	Species	Peptide accuracy (%)
Bacillus	98.01	Bacillus	97.61
C. bact	95.82	C. bact	96.10
Honeybee	98.19	Honeybee	97.94
Human	98.44	Human	98.83
M. mazei	97.86	M. mazei	98.16
Mouse	98.15	Mouse	97.61
Rice bean	96.90	Rice bean	88.12
Tomato	98.20	Tomato	97.90
Yeast	97.94	Yeast	97.70
Average	97.90	Average	96.28

3 Peptide accuracy–coverage curves

Figure S3 presents peptide accuracy–coverage curves for π-PrimeNovo, RankNovo, and ScopeNovo (Setting 1 and Setting 2). All predictions are ranked in descending order according to π-PrimeNovo model confidence, and coverage is progressively increased by including predictions from highest to lowest confidence; at each coverage level, peptide accuracy is computed on the retained subset. Under this protocol the curve reflects whether reranking can correct predictions on spectra with progressively lower π-PrimeNovo confidence. On 9-species-V1, ScopeNovo (Setting 1) consistently attains the highest accuracy–coverage curve across nearly all species, while ScopeNovo (Setting 2) remains above the base π-PrimeNovo model for most of the coverage range, with particularly clear gains on *C. bact*, *Honeybee*, *M. mazei*, *Mouse*, and *Tomato*. A similar trend holds on 9-species-V2. Importantly, these gains persist as coverage approaches 1.0, where the curves converge to the full-dataset accuracies reported in our paper.

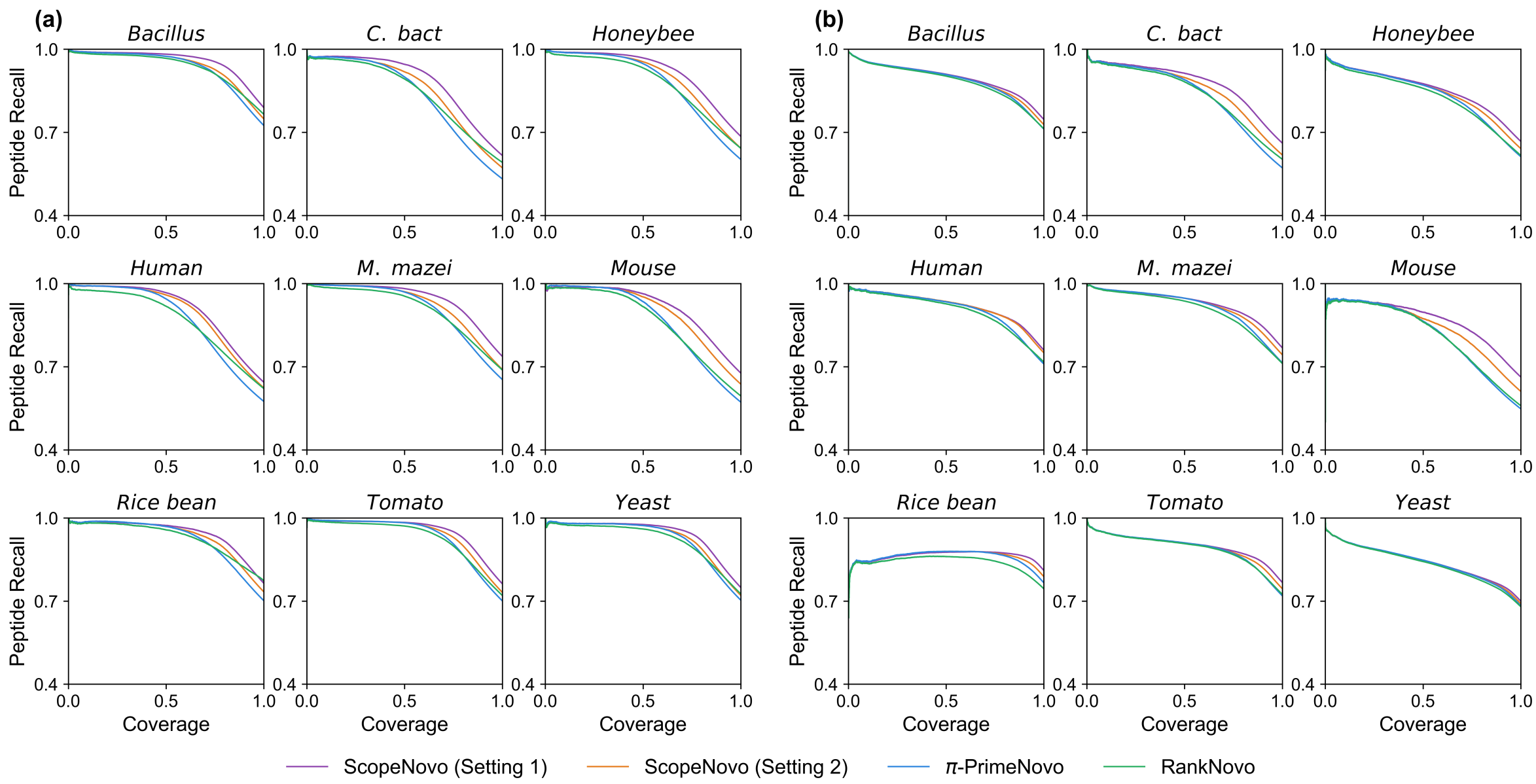

Figure S3: Peptide accuracy–coverage curves of π-PrimeNovo, RankNovo, and ScopeNovo (Setting 1 and Setting 2). Curves are plotted by ranking predictions according to π-PrimeNovo model confidence on the (a) 9-species-V1 and (b) 9-species-V2 datasets.

4 Inference time with different candidate set sizes

The inference time required by ScopeNovo depends on the number of initial candidate peptide sequences K generated by the original transformer model. The inference time remains stable across $K \in \{20, 50, 100\}$. See Fig. S4.

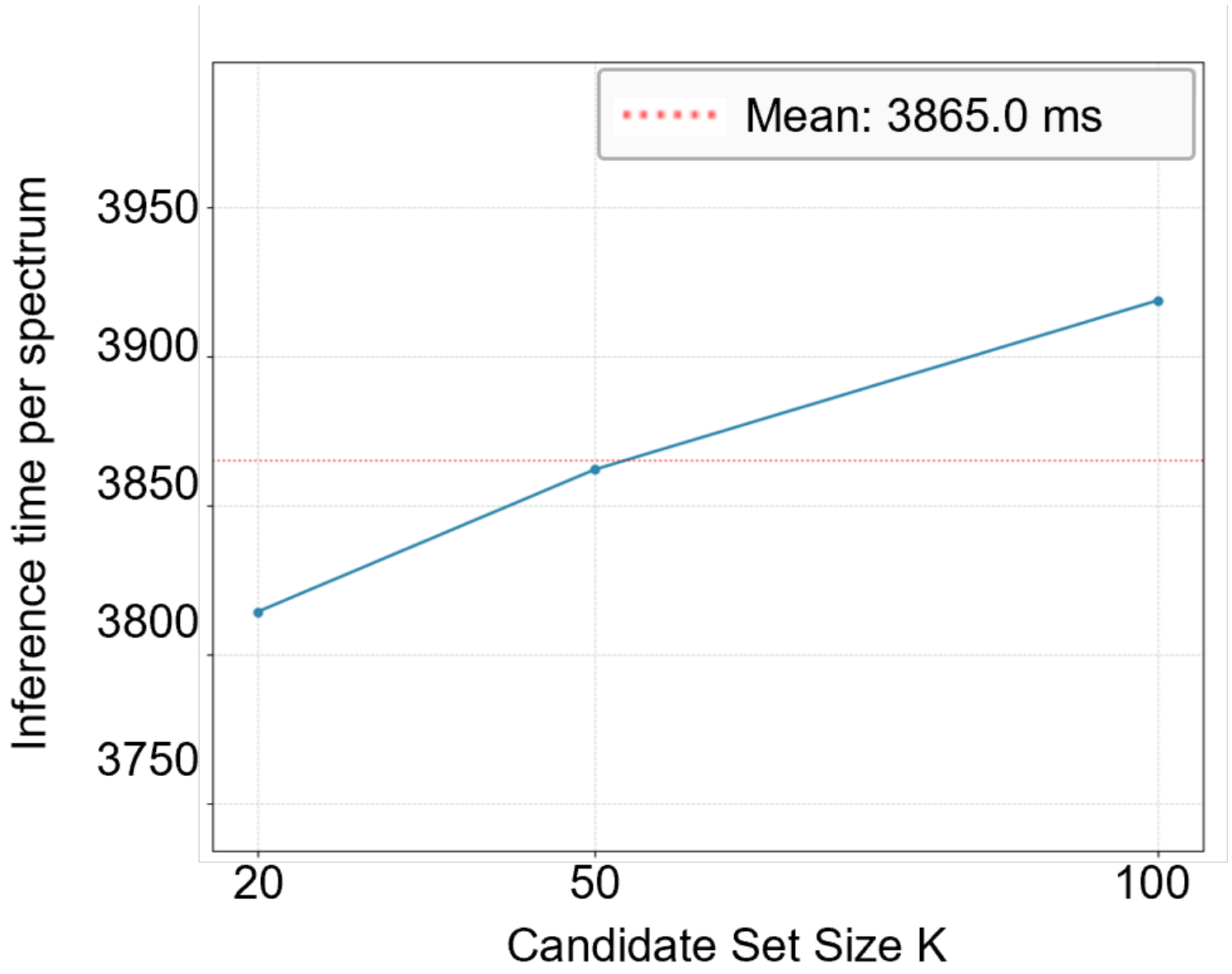

Figure S4: Inference time per spectrum of ScopeNovo across different candidate set sizes K.

5 Understanding the impact of peaks

We define a peak as theoretical if it can be explained by a b/y fragment ion of the ground-truth peptide sequence within a 0.1 Da mass tolerance, and as non-theoretical (noise) otherwise. This experiment is conducted on the Mouse test split of 9-species-V1. Note that mass spectra have lots of noise peaks as well as peaks corresponding to theoretical peaks of the peptide sequence. To understand how these two kinds of peaks affect the de novo sequencing process, we remove some percentages of non-theoretical and theoretical peaks, respectively, and observe the corresponding peptide accuracies. Fig. S5 (a) shows the results for removing non-theoretical peaks. The blue and red bars represent the performances of π-PrimeNovo and ScopeNovo, respectively. The green bars represent the performance of a modified ScopeNovo version, where instead of using latent spectrum-spectrum similarity, we use the modified cosine similarity. We can see that when non-theoretical peaks are removed from 10% to 50%, the performances of the three methods slightly decrease. This shows that the non-theoretical peaks have minor impact on the performance. Fig. S5 (b) shows the results for removing theoretical peaks. We can see that when 10% of theoretical peaks are removed, the performances of all the three methods decrease slightly. When 20% of theoretical peaks have been removed, the performances of the three methods drop dramatically.

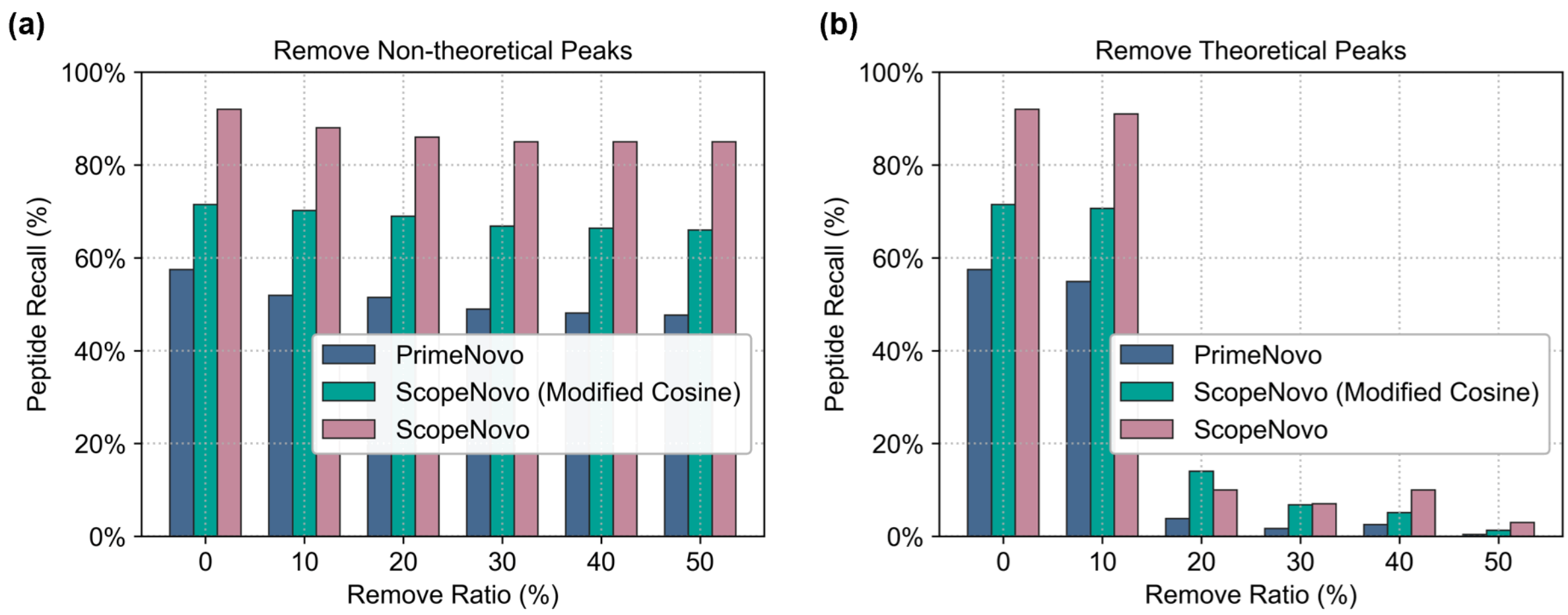

Figure S5: Effect of structured peak removal on peptide accuracy. We report peptide-level accuracy when removing the top-intensity p% peaks from (a) unmatched (non-theoretical) peaks or (b) matched theoretical b/y peaks, comparing PrimeNovo, ScopeNovo (Modified Cosine), and ScopeNovo.

6 Generalization to a held-out instrument and study

To further evaluate the generalizability of ScopeNovo beyond tryptic data, we evaluate ScopeNovo and other baseline models on PXD009737-GluC, a non-tryptic *Human* dataset generated by Sun et al. and reanalyzed by π-MSNet. PXD009737-GluC is generated using GluC digestion and contains longer peptide sequences. It therefore differs substantially from the training data of π-PrimeNovo and the nine-species benchmarks. As shown in Table S2, ScopeNovo achieves the highest peptide accuracy 44.70% among all evaluated methods, outperforming RankNovo (third best) and Instanovo (second best) by 3.03% and 1.97%, respectively. ScopeNovo also improves the peptide accuracy of the base π-PrimeNovo model from 40.77% to 44.70% and the amino-acid precision from 57.93% to 58.66%.

Table S2: Comparison of de novo peptide sequencing methods on PXD009737-GluC.

Method	AA Precision (%)	Peptide Accuracy (%)
AdaNovo	26.75	11.10
Casanovo	48.24	27.68
CasanovoV2	49.98	28.39
Instanovo	**61.55**	42.73
ContraNovo	56.26	37.01
PrimeNovo	57.93	40.77
RankNovo	59.85	41.67
ScopeNovo	58.66	**44.70**

7 Source attribution of successful corrections

An important question is whether ScopeNovo's corrections are driven by *empirical* spectral-library matches or by *predicted* (simulated, e.g., Prosit-generated) spectra. We categorize every test spectrum whose prediction is *corrected* by ScopeNovo (the base model's top-1 is wrong, but ScopeNovo's reranked top-1 is correct) according to the source of the highest-scoring reference spectrum used in the reranking: an *empirical* reference retrieved from the peptide-spectrum library, or a *predicted* reference generated by Prosit. Table S3 reports the breakdown on the Mouse test set, a representative Setting 1 case using the external peptide–spectrum library. Under Setting 1 the majority of corrections are attributable to empirical-library matches, while a non-negligible fraction rely on Prosit-predicted spectra; the latter are precisely the cases in which the correct peptide is absent from the empirical library and can only be recovered through simulation, which is the route through which novel-peptide candidates are reranked.

Table S3: Sources of reference spectra associated with successful ScopeNovo corrections on the Mouse test set.

Reference spectrum source	Corrections	Proportion
Peptide-Spectrum Library	2,443	96.52%
Spectrum predictor (Prosit)	88	3.48%
